# *Aralia armata* (Wall.) Seem Improves Intimal Hyperplasia after Vascular Injury by Downregulating the Wnt3*α*/Dvl-1/*β*-Catenin Pathway

**DOI:** 10.1155/2021/6682525

**Published:** 2021-07-12

**Authors:** Xiangpei Zhao, Jinchang Huang, Zhenyu Mo, Jiangcun Wei, Chuanmei Zhong, Hongli Teng

**Affiliations:** ^1^Department of Technology, Guangxi International Zhuang Medicine Hospital, Nanning 530201, China; ^2^Department of Academic Affairs, Ruikang Clinical Medical College, Guangxi University of Traditional Chinese Medicine, Nanning 530200, China

## Abstract

The aim of the study is to examine the mechanism of *Aralia armata* (Wall.) Seem (AAS) in improving intimal hyperplasia after vascular injury in rats. Rats with femoral artery injury were randomly divided into three groups: the model group, AAS low-dose group (40 mg/kg), and AAS high-dose group (80 mg/kg). The sham operation group was used as a control group. HE staining was used to observe the changes in femoral artery vessels. Immunohistochemistry was adopted to detect *α*-SMA, PCNA, GSK-3*β*, and *β*-catenin proteins in femoral artery tissue. The CCK-8 test and wound healing assay were employed to analyze the effect of AAS on proliferation and migration of vascular smooth muscle cells (VSMCs) cultured *in vitro*. Western blotting (WB) and polymerase chain reaction (PCR) assays were used to evaluate the molecular mechanism. AAS reduced the stenosis of blood vessels and the protein expressions of *α*-SMA, PCNA, GSK-3*β*, and *β*-catenin compared to the model group. In addition, AAS (0-15 *μ*g/mL) effectively inhibited the proliferation and migration of VSMCs. Moreover, the results of WB and PCR showed that AAS could inhibit the activation of *β*-catenin induced by 15% FBS and significantly decrease the expression levels of Wnt3*α*, Dvl-1, GSK-3*β*, *β*-catenin, and cyclin D1 in the upstream and downstream of the pathway. AAS could effectively inhibit the proliferation and migration of neointima after vascular injury in rats by regulating the Wnt/*β*-catenin signaling pathway.

## 1. Introduction

Atherosclerotic diseases are the leading cause of death in both developed and undeveloped nations. Their incidence rate is increasing every year. Atherosclerosis can start at a very young age [1]. Percutaneous coronary intervention (PCI) is considered the most common treatment for severe coronary atherosclerotic heart disease. During this procedure, a small structure, also known as a stent, is used to open narrow blood vessels.

Restenosis (RS) is an important factor restricting the long-term development of PCI [2, 3]. The pathological process of restenosis involves many factors and multiple pathways; still, the exact mechanism remains unclear. So far, several theories have been proposed, including the theory of wound repair, which argues that restenosis is a pathological repair response of blood vessels to traumatic treatment [4]. Neointima is a vital link in RS. After the vascular endothelium is damaged, vascular smooth muscle cells (VSMCs) undergo a phenotypic transformation, migrate from media to intima, and excessively proliferate, resulting in a large accumulation of VSMCs in vascular intima and narrowing of the lumen [5, 6]. Therefore, new potential drugs should be able to inhibit the abnormal proliferation of VSMCs and block or slow down restenosis, which has become one of the priorities in the research and development of anticardiovascular drugs.

So far, few studies have reported on the effect of certain drugs and restenosis [7, 8]. Moreover, some traditional medicines, such as *Camellia japonica* [9], docetaxel [10], *Alpinia officinarum* Hance [11], *Salvia miltiorrhiza* [12], and *Panax notoginseng* [13], which can promote blood circulation and remove blood stasis, have an inhibitory effect on VSMC proliferation. Previous studies have also shown that emodin [14] and chebulinic acid [15] can inhibit collagen synthesis by vascular smooth muscle cells. Some traditional medicine with anti-inflammatory effects, such as chicoric acid [16] and *Andrographis paniculata* [17], has also shown certain effect on the prevention of restenosis after vascular injury.

*Aralia armata* (Wall.) Seem (AAS) is a medicinal and edible plant primarily found in the Guangxi Zhuang Autonomous Region. Its tender buds and stems are edible, with unique flavor and fragrance. This plant is rich in amino acids and has more than 16 kinds of inorganic nutrient elements required by the human body [18]. It participates in liver protection and regulates the growth of the human body. It also has a good curative effect on acute and chronic inflammation and various neurasthenia diseases [19, 20]. Doctors often use its root bark to treat cardiovascular diseases, chronic nephritis, prostatitis, edema, and rheumatic arthralgia. So far, few studies have investigated the root bark of AAS. Some scholars found that the chemical composition of this plant is rich in saponins [21]. Relevant studies have also confirmed that the saponins from the plants in the same genus have anti-inflammatory [22, 23], anticancer [24–26], antiatherosclerosis [27–30], and antidiabetes [31, 32] effects. However, so far, no study has reported on the effect of AAS (root bark) on intimal hyperplasia after a vascular injury.

The aim of this study was to examine the mechanism of AAS in improving intimal hyperplasia after vascular injury in rats. In addition, we also explored the effect of AAS on the proliferation and migration of VSMCs induced by 15% FBS and evaluated its molecular mechanism. Our results showed that AAS has an inhibitory effect on the formation of neointima induced by femoral artery injury.

## 2. Materials and Methods

### 2.1. Preparation of *Aralia armata* (Wall.) Seem (AAS)

The *Aralia armata* (Wall.) Seem was collected in Guangxi Province and was authenticated by the authors (Prof. Richun Lan). Specimens of these materials were deposited in the herbarium, Guangxi International Zhuang Medicine Hospital, China. The dried root bark of *Aralia armata* (Wall.) Seem was taken and pulverized into a coarse powder. Then, 200 g was mixed with 2000 mL of 80% ethanol and refluxed. The extract was obtained through vacuum concentration. Then, 500 mL of double-distilled water was added for full dissolution, and the solution was moved into the separating funnel; 500 mL of petroleum ether was added for extraction. Consequently, a low aqueous phase solution was taken from a liquid separation funnel. The volume of the water phase was calculated, and an equal volume of chloroform was added for well mixing. The upper solution was taken and put in the separating funnel, and an equal volume of n-butanol was added for slightly shaking. The upper solution was taken, and n-butanol was extracted twice. The extracts were combined and rotated for evaporation to recover the solvent, steamed dry in a water bath to obtain 14 g of dry extract, and put into a brown glass bottle for later use. AAS powder was dissolved in normal saline during administration.

### 2.2. Preliminary Identification of AAS Saponins

The chemical constituents of AAS were preliminarily identified by ultra-high-performance liquid chromatography-quadrupole-electrostatic field orbital trap high-resolution mass spectrometry (UPLC-Q-Orbitrap HRMS). Briefly, 1.0 mg AAS powder was dissolved in 1.0 mL methanol : water (8 : 2, *V* : *V*) and treated with ultrasound (40 kHz, 150 W) for 30 min using a 0.22 *μ*m microporous membrane filtration. Compounds were separated on a Welch Ultimate Polar RP-C18 column (2.1 mm × 150 mm, 1.8 *μ*m). The mobile phase was 0.1% formic acid solution and 0.1% formic acetonitrile solution; the flow rate was 0.30 mL/min, and the column temperature was 35°C. HRMS was performed using an electrospray ion source (ESI) and scanned in a positive ion mode using full scan/data-dependent secondary scan (full mass/dd-MS2). CD2.1 software (Thermo Fisher) that was linked to the mzCloud, mzVault, and ChemSpider network database was used to analyze the data based on accurate molecular mass, retention behaviors, and characteristic ion fragmentation of the compounds, as well as literature information and relevant reference materials.

### 2.3. Rat Femoral Artery Injury Model and Grouping

Thirty-two SD rats, 6-8 weeks old and weighing 180-220 g, were obtained from Hunan SJA Laboratory Animal Co., Ltd., China. All the animals were housed in an environment with a temperature of 22 ± 1°C, relative humidity of 50 ± 1%, and a light/dark cycle of 12/12 h. Rats were given ad libitum access to food and water for 1 W. All animal studies, including the animal euthanasia procedure, were done in compliance with the regulations and guidelines of Guangxi International Zhuang Medicine Hospital institutional animal care and conducted according to the AAALAC and the IACUC guidelines (official permission letter no. 20190518-15).

Twenty-four rats were then randomly selected to establish the femoral artery exfoliation endothelium model. Briefly, rats were anesthetized by isoflurane inhalation. Then, a lower limb skin was cut, and femoral artery branches were dissociated and cut. A 1.00 mm diameter Micro Therapeutics (Inc.) was then inserted into the femoral artery and pulled back and forth 3 times to cause arterial endothelium exfoliation. After the operation, the arterial incision was ligated, and the skin was sutured [33]. In the sham operation group, only skin and artery incisions were made, and no interventional guidewire was inserted. The rats that were successfully operated were randomly divided into three groups: the model group, AAS low-dose group (40 mg/kg), and AAS high-dose group (80 mg/kg). All groups were continuously given intragastric administration for 4 W and the drug group was given the corresponding liquid medicine, while the normal group and the model group were given the same volume of normal saline (10 mL/kg) once a day. During the intervention period, the general state of rats in each group was monitored, including body mass, eating and drinking, defecation and urination, and hair.

### 2.4. Determination of the Vascular Infarction Ratio

After the operation, the animals were anesthetized and perfused with neutral formalin under constant pressure systemic circulation for 15 min. The femoral artery was cut off, washed with normal saline, soaked in formalin, and fixed for 24 h. The femoral artery and its surrounding tissues were cut into 2 mm long segments, dehydrated with conventional alcohol, and embedded in paraffin to make 5 *μ*m thick blood vessel continuous cross-sectional sections. The tissue sections were stained with HE, and the vascular morphology was observed under a microscope. The perimeter of the vascular lumen was measured by using Image-Pro plus 6.0 image analysis software, and the equivalent circular area of the bleeding tube lumen was converted. Then, the vascular intima area was measured, after which the vascular infarction ratio was calculated using the following formula [34, 35]: intima area/equivalent circular area of the vascular lumen × 100%. The single-blind method was used for the measurement, and the measurer was unrelated to this experiment.

### 2.5. Immunohistochemistry

Immunohistochemical staining was carried out using a SABC method. Anti-*α*-SMA, PCNA, GSK-3*β*, and *β*-catenin were used as primary antibodies, respectively, and other operations were carried out according to the kit instructions [36]. The new DAB was used to develop color, and the staining degree was controlled under a microscope. PBS was adopted as a negative control instead of the primary antibody. Five different fields of view were randomly selected from each section. The area of interest (AOI) and integrated optical density (IOD) were measured by using Image-Pro Plus 6.0 software, and the average IOD/AOI was calculated to evaluate the degree of regional staining.

### 2.6. Cell Culture

Referring to a previous study [37], the rat aorta was isolated and cultured by enzymatic digestion. Briefly, animals were anesthetized, and their chest was opened. The aorta was flushed clean by perfusion from the left ventricle; the perivascular fat and connective tissue were removed. The thoracic aorta was then taken out and put in a mixed enzyme digestion solution prepared with Hanks balanced salt (collagenase II 1 mg/mL, elastase 0.744 U/mL, soybean trypsin inhibitor 1 mg/mL, and BSA 2 mg/mL in HBSS). After incubation at 37°C for 15 min, the outer membrane was carefully detached with tweezers. The inner membrane was gently wiped off with cotton swabs; the obtained media tissue was cut into fragments. A freshly mixed enzyme digestion solution was added for incubation at 37°C for 2-3 h. Then, the digested tissue was gently smashed into a cell suspension and centrifuged at 1000 rpm for 5 min. Consequently, cells were collected. DMEM culture solution containing 10% FBS was added for cell resuspension, inoculated in a culture flask, and digested with 0.125% trypsin/EDTA. The cultured smooth muscle cells were morphologically observed under an inverted phase-contrast microscope. Immunocytochemical staining was performed with an anti-*α*-SMA monoclonal antibody and Alexa Fluor 594-labeled secondary antibody. The number of positive cells was observed under a fluorescence microscope. The 5th-9th passage cells were used for experiments.

### 2.7. Cell Proliferation Assay

VSMCs in the logarithmic growth phase were inoculated into a 96-well culture plate at a density of 1.0 × 10^4^ cells per well and starved for 24 h. The VSMCs were divided into the following groups: normal control group, AAS dose group (1.875, 3.75, 7.5, 15, and 30 *μ*g/mL), 15% FBS group, and AAS dose group (1.875, 3.75, 7.5, and 15 *μ*g/mL)+15% FBS group. All cells were treated for 12 h, 24 h, and 36 h, respectively. After the treatment, 10 *μ*L CCK-8 solution was added to each well; the solution was then shaken and incubated in an incubator for an additional 2 h. The optical density (OD) of each group was measured at 450 nm wavelength by using an enzyme labeling instrument: OD value = determination well − blank well. The experiment should be repeated at least three times.

### 2.8. Wound Healing Assay

VSMCs were inoculated into a 6-well plate (3.0 × 10^5^ cells/well). After adhering to the wall, the cells were lightly scribed with a 100 *μ*L nozzle and washed twice with PBS. According to the experimental results of 2.6, two doses (7.5 and 15 *μ*g/mL) of AAS with low cytotoxicity and effective inhibition of 15% FBS-induced proliferation were used to treat cells for 24 h. The scratches of each well were observed and photographed under a microscope (Olympus, Japan, 100x), and the average width of scratches was recorded at 0 h and 24 h, respectively. Scratch healing was calculated as follows: scratch healing = initial 0 h average scratch width − 24 h average scratch width [38]. The experiment should be repeated at least three times.

### 2.9. RT-qPCR Detection

The total RNA of VSMCs was routinely extracted using the TRNzol Universal Reagent (purchased from TIANGEN), and *D*(*λ*)_260_/*D*(*λ*)_280_ was determined by using an infinite TECAN enzyme labeling instrument (model: M200PRO). If *D*(*λ*)_260_/*D*(*λ*)_280_ was 1.8-2.0, it was stored for later use. A total of 1 *μ*g of total RNA was taken to synthesize cDNA (Fast Reverse Transcription Master Mix from BioTech Inc.). Residual DNA was removed at 42°C for 5 min after which reverse transcription was performed at 42°C for 15 min; reverse transcriptase was inactivated at 95°C for 5 min. A total of 1.2 *μ*L reverse transcription reaction product was used for the real-time fluorescence PCR reaction. According to the SYBR Green qPCR Mix kit (purchased from BioTech Inc.), a two-step thermal cycle was adopted. The reaction conditions were as follows: predenaturation at 95°C × 3 min for 1 cycle, denaturation at 95°C × 10 s, and annealing at 60°C × 30 s, and repeated for 40 cycles. All samples were added to 96-well PCR plates, each sample was repeated for 3 wells, and all reactions were carried out in a Roche LightCycler Sequence Detection System. The primer sequence is shown in Table 1. The relative quantitative method adopted the comparative Ct method, GAPDH was used as an internal reference, the △Ct (Ct_target_ − Ct_internal reference_) method was used for relative quantitative analysis, and the 2^−△△Ct^ was used as the relative expression amount of target RNA [39].

### 2.10. Western Blotting

After treatment, cells were collected. The protein was extracted according to the instructions of the protein extraction kit. The protein concentration was measured using the BCA method and stored at -80°C for later use. Proteins were separated on 10% SDS-PAGE. Immunoblotting was used to transfer samples to the PVDF membrane, which was incubated in 5% skimmed milk powder solution overnight at 4°C. The membrane was then incubated with Wnt3*α*, *β*-catenin, Gsk-3*β*, Dvl-1, cyclin D1, and GADPH antibodies at room temperature for 4 h, after which it was washed for 3 times in TBST and incubated again with the corresponding horseradish peroxidase-labeled secondary antibody. Samples were analyzed using a chemiluminescent protein detection method and Image software [40].

### 2.11. Statistical Analysis

SPSS 19.0 was used for statistical treatment. The value was expressed as mean ± standard deviation. The independent-sample *t*-test was used when the two-pair comparison was in accordance with the normality. In the comparison of multiple groups, the LSD-*t* or Dunnett *t*-test was used when the variance was homogeneous and in conformity with the normality. The nonparametric *t*-test was used when the variance was not in conformity with the normality. Dunnett's T3 or Tamhane's T2 test was used when the variance was in conformity with the normality, but not in conformity with the variance. *P* ≤ 0.05 was considered statistically significant.

## 3. Results

### 3.1. Several Saponins in AAS

**Figure 1:**
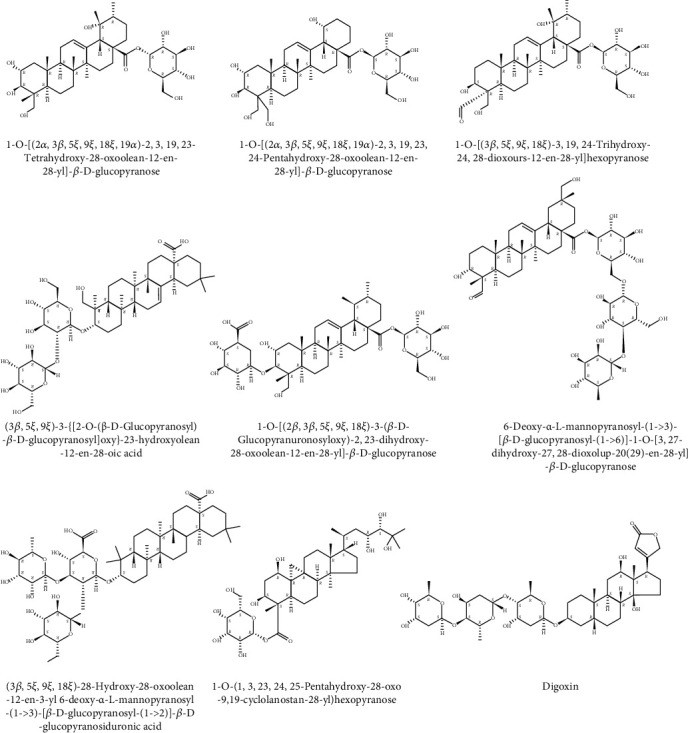
Structures of several saponins in AAS.

AAS was detected by UPLC-Q-Orbitrap HRMS and compared with related databases. The structural formulas of eight oleanolic acid triterpenoid saponins and one cardiotonic glycoside were identified (Figure 1).

### 3.2. AAS Improves Intimal Hyperplasia Caused by Femoral Artery Mechanical Injury

**Figure 2:**
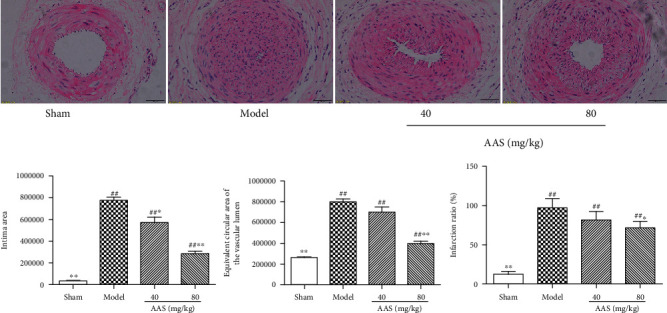
AAS improves intimal hyperplasia in the femoral artery injury rat model. Representation of the arterial vascular walls in four groups by using HE staining (magnification ×400). Statistical analysis of the intimal area, the equivalent circular area of the vascular lumen, and the vascular infarction ratio in the four groups. All the data are represented as mean ± sd. ^∗^*P* < 0.05 and ^∗∗^*P* < 0.01 compared with the model group; ^#^*P* < 0.05 and ^##^*P* < 0.01 compared with the sham group.

Classical histological HE staining analysis showed that the model group had noticeable changes in vascular structure and narrowed vascular lumen compared with the sham operation group. As shown in Figure 2, severe intimal hyperplasia was observed in the model group, with incomplete intimal repair, discontinuous internal elastic plates, the rough inner surface of the lumen, and migration and proliferation of a large number of smooth muscle cells from the media to the intima. In addition, obvious thickening of the intima, accumulation of a large amount of matrix, and obvious narrowing of the lumen were observed. As expected, these pathological changes were significantly reduced, and the internal and external elastic plates were relatively intact in the administration group.

Image-Pro Plus 6.0 software was used to further analyze the vascular morphology. First, the intimal area and the equivalent circular area of vascular lumen were measured, and the vascular infarction ratio was obtained by dividing them. The results showed that the proportion of vascular infarction was significantly increased in the model group compared with the sham operation group (12.37% vs. 97.14%, *P* < 0.01). The proportion of vascular infarction in the AAS high- and low-dose groups was 71.48% and 81.75%, respectively; there was a significant difference between the high-dose group and the model group (*P* < 0.05). These data showed that AAS can inhibit neointimal hyperplasia induced by vascular injury.

### 3.3. AAS Decreases the Cell Proliferation Rate of Injured Vessels

**Figure 3:**
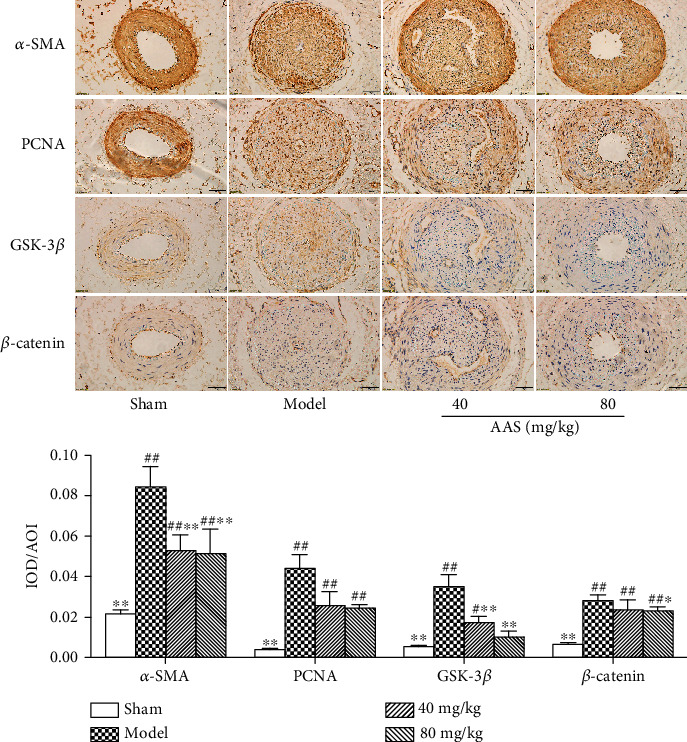
AAS improves intimal hyperplasia in the femoral artery injury rat model. Representation of the arterial vascular walls in four groups by using semiquantitative immunohistochemistry (magnification ×400). Statistical analysis of the expression of *α*-SMA, PCNA, GSK-3*β*, and *β*-catenin in the injured artery. All the data are represented as mean ± sd. ^∗^*P* < 0.05 and ^∗∗^*P* < 0.01 compared with the model group; ^#^*P* < 0.05 and ^##^*P* < 0.01 compared with the sham group.

Previous studies have shown that VSMCs differentiated from the contractile type to the synthetic type promote the occurrence and development of vascular remodeling through cell migration and proliferation, resulting in the proliferation of vascular intima [37]. In this study, we used *α*-SMA as a marker protein of the VSMC contractile phenotype to analyze the neointima's damage (Figure 3). Compared with the sham operation group, the expression of *α*-SMA in the neovascularization intima of the model group rats was significantly reduced, indicating that VSMCs in the neovascularization intima of the model group rats underwent a phenotypic transformation from the contractile type to the synthetic type. Compared with the model group, the expression of *α*-SMA in the AAS group increased, thus suggesting that AAS can inhibit the transformation of VSMCs from the contractile phenotype to the synthetic type in the damaged neointima.

PCNA is an essential biological index reflecting cell proliferation [41, 42]. In order to investigate the antiproliferation effect of AAS, the expression of PCNA in damaged blood vessels was detected. No PCNA staining-positive cells were found in the sham operation group (Figure 3). Smooth muscle cells in the neointima of the model group showed diffuse thickening, high expression of PCNA-positive cells, and deep staining. The thickening degree of the neointima of the AAS group was significantly lower than that of the model group, and only a small number of PCNA-positive cells were found in the neointima near the official cavity. In addition, the staining degree was lighter.

The immunohistochemical semiquantitative analysis showed that the average optical density of the AAS group was significantly different from that of the model group (*P* < 0.05), which suggested that AAS inhibited the proliferation of intimal cells after vascular injury, thus reducing lumen stenosis.

### 3.4. AAS Inhibits VSMC Proliferation Induced by Serum

**Figure 4:**
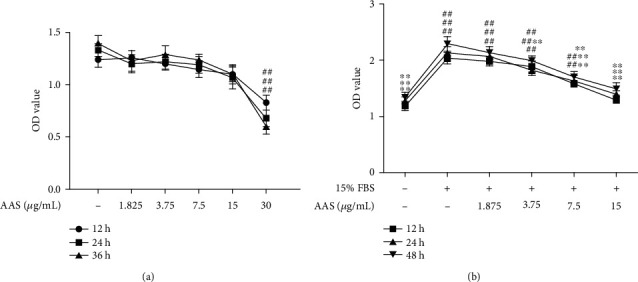
The effect of AAS on VSMC proliferation. (a) The cells were incubated with AAS (1.875, 3.75, 7.5, 15, and 30 *μ*g/mL) for 12, 24, and 48 h. (b) The cells were incubated with 15% FBS+AAS (1.875, 3.75, 7.5, and 15 *μ*g/mL) for 12, 24, and 48 h. Results are shown as means ± sd. ^∗^*P* < 0.05 and ^∗∗^*P* < 0.01 compared with control (15% FBS alone).

Overgrowth of VSMCs is considered to be the main factor leading to restenosis of vascular injury. In this study, CCK-8 detection was carried out to determine the inhibitory effect of AAS on the proliferation of VSMCs. First, we detected the cytotoxicity of AAS to normally growing VSMCs. As shown in Figure 4(a), no obvious cytotoxicity was observed, except for a 30 *μ*g/mL dose group. Therefore, we chose to detect the inhibitory effect of the AAS 1.875, 3.75, 7.5, and 15 *μ*g/mL on the proliferation of VSMCs induced by 15% FBS (Figure 4(b)). Our data indicated that AAS could effectively inhibit the proliferation of VSMCs induced by serum in a dose-dependent and time-dependent manner.

### 3.5. AAS Slows Down the Migration of Monolayer Cells

**Figure 5:**
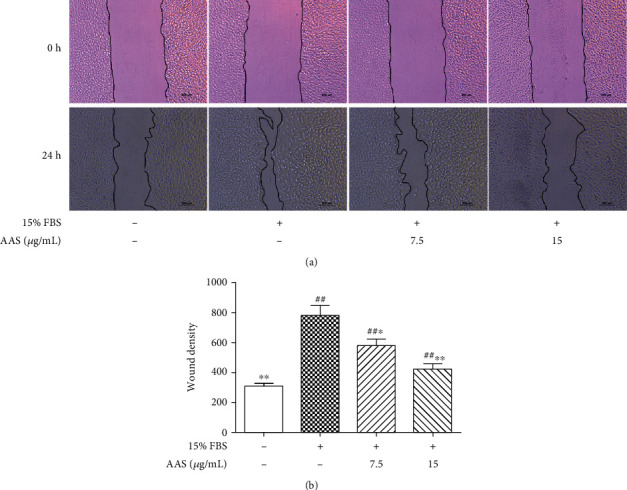
The effect of AAS on VSMC migration. (a) A wound healing assay was used to evaluate the migration of VSMCs. (b) Wound density was quantified as a percentage of the initial wound area that had been recovered with VSMCs. Results are shown as means ± sd. ^∗^*P* < 0.05 and ^∗∗^*P* < 0.01 compared with control (15% FBS alone).

The effect of AAS on VSMC migration was evaluated using a wound healing test. As shown in Figure 5(a), AAS significantly inhibited the migration of serum-induced VSMCs 24 hours after injury. The scratch healing degrees of 7.5 *μ*g/mL and 15 *μ*g/mL AAS were 578.85 and 420.97, respectively, and were significantly different from those of the model group (Figure 5(b)).

### 3.6. AAS Inhibits Wnt3*α*/Dvl-1/*β*-Catenin Pathway-Related Gene Expression

**Figure 6:**
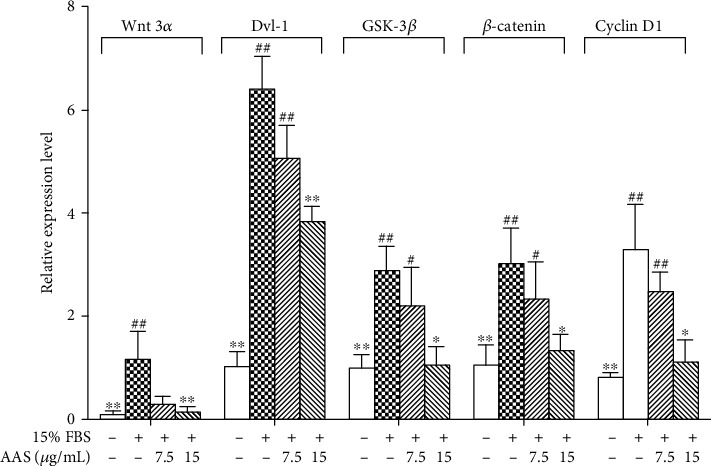
AAS regulates 15% FBS-induced expressions of Wnt3*α*/Dvl-1/*β*-catenin signaling molecules in VSMCs. The Wnt3*α*, *β*-catenin, Gsk-3*β*, Dvl-1, and cyclin D1 expression was evaluated using real-time quantitative PCR (RT-qPCR). Data is represented as means ± sd. ^∗^*P* < 0.05 and ^∗∗^*P* < 0.01 compared with control (15% FBS alone).

RT-qPCR results showed that the AAS high-dose group (15 *μ*g/mL) significantly inhibited the high expression of Wnt signaling pathway key protein molecules (Wnt3*α*, Dvl-1, Gsk-3*β*, *β*-catenin, and cyclin D1) of mRNA induced by 15% FBS in vascular smooth muscle cells (Figure 6).

### 3.7. AAS Inhibits Wnt3*α*/Dvl-1/*β*-Catenin Pathway-Related Protein Expression

**Figure 7:**
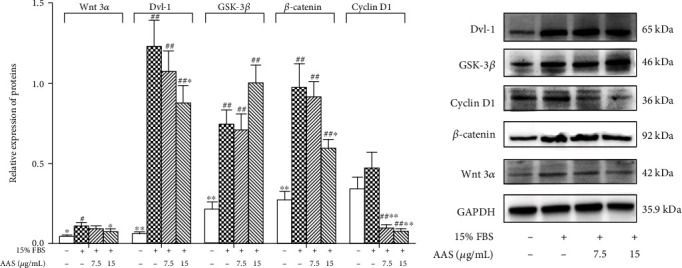
AAS regulates 15% FBS-induced expressions of Wnt3*α*/Dvl-1/*β*-catenin signaling molecules in VSMCs. Wnt3*α*/Dvl-1/*β*-catenin signaling protein expression was determined by Western blotting. Data is represented as means ± sd. ^∗^*P* < 0.05 and ^∗∗^*P* < 0.01 compared with control (15% FBS alone).

The results of Western blot analysis (Figure 7) showed that AAS could inhibit the overexpression of Wnt signaling pathway proteins (Wnt3*α*, *β*-catenin, Gsk-3*β*, Dvl-1, and cyclin D1) induced by 15% FBS, especially the overexpression of *β*-catenin, cyclin D1, and Gsk-3*β*. This was further confirmed by immunohistochemistry. As shown in Figure 7, AAS can inhibit the expression levels of *β*-catenin and Gsk-3*β* in damaged blood vessels, with a significant difference compared with the model group (*P* < 0.05).

## 4. Discussion

The exact mechanism of restenosis after angioplasty remains unclear. Most scholars believe that the proliferation and migration of media smooth muscle cells are essential for restenosis after vascular injury. The abnormal proliferation of VSMCs has an important role in the occurrence and development of vascular remodeling. After vascular injury, the inflammation, proliferation, and migration of vascular smooth muscle cells increase, and the expression of smooth muscle markers decreases, thus leading to intimal hyperplasia [43, 44]. The proliferation, migration, and cell-extracellular matrix (ECM) adhesion of VSMCs are related to intimal hyperplasia of some vascular lesions, including restenosis and atherosclerosis [45, 46]. Therefore, regulating the growth of VSMCs is of critical therapeutic significance [23].

In this study, we demonstrated for the first time that the AAS could reduce neointimal hyperplasia after vascular injury. We found that AAS could significantly reduce the formation of the new intima after arterial injury (Figure 2); the proportion of vascular infarction was significantly lower than that of the model control group (Figure 2). In order to prove that the AAS has an effective inhibitory effect on the growth of VSMC, we detected the expression of *α*-SMA and PCNA in damaged blood vessels by immunohistochemistry (Figure 3) and examined VSMC proliferation using a CCK-8 assay (Figure 4) and wound healing assay (Figure 5). All these data suggested that the AAS participates in improving restenosis after vascular injury.

The regulation of the Wnt signaling pathway in vascular restenosis is considered a topic of interest among scientists. Studies on tissue embryo development showed that the Wnt signaling pathway is activated in the early formation stage of vascular smooth muscle cells (VSMCs) [47, 48]. Lv et al. found that inhibition of the Wnt classical pathway reduces cyclin D1 expression and VSMC proliferation [49]. Moreover, Williams et al. showed that the *β*-catenin signal is activated after carotid artery injury in rats [50]. Other researchers have found that upstream signal molecules, such as Wnts/Fzds, Dvl, and GSK-3*β*, participate in smooth muscle cell proliferation after vascular damage [51–55]. The above findings suggest that the Wnt/*β*-catenin signaling pathway may participate in vascular development or proliferation after injury. To further study the mechanism of inhibition of VSMC proliferation and migration by AAS, we detected the expression of upstream and downstream signal molecules in the Wnt signaling pathway. The obtained results showed that the mRNA and protein expressions of Wnt3*α*, GSK-3*β*, Dvl-1, and *β*-catenin increased when VSMCs were induced to proliferate by serum, and the mRNA and protein expressions of Wnt3*α*, GSK-3*β*, Dvl-1, and catenin simultaneously decreased when FBS-induced cell proliferation was inhibited by AAS (Figures 6 and 7). The downregulation of the expression levels of *β*-catenin and Gsk-3*β* by the AAS has been further confirmed in animal experiments (Figure 3).

The content of cyclin changes rapidly with the circulation of the cell cycle. Cells in the quiescent state do not express cyclin D1. When growth factors stimulate cells, cyclin D1 is expressed, which in turn binds to cyclin-dependent kinase to phosphorylate, thus entering the cell proliferation state. Cyclin D1 is the downstream target gene of Dvl-1. Our *in vitro* cell experiment showed that AAS inhibited the high expression of serum-induced Wnt signaling molecules and downregulated the expression of cyclin D1 (Figures 6 and 7). Therefore, we believe that the change of cyclin D1 further supports AAS to inhibit intimal proliferation after vascular injury by downregulating the Wnt signaling pathway.

*Aralia armata* (Wall.) Seem is widely used as a traditional medicine to treat cardiovascular diseases, chronic nephritis, prostatitis, edema, rheumatic arthralgia, and other diseases. This study further suggested that the AAS has a strong cardiovascular protection effect and can be used as a good candidate to prevent and treat cardiovascular diseases.

The main limitation in this study is the lack of more information on the *Aralia armata* (Wall.) Seem. Recently, Hui et al. have isolated various oleanane triterpenes from the root of *Aralia armata* (Wall.) Seem and evaluated their cytotoxicity [21]. In this article, eight oleanolic acid triterpenoid saponins and one cardiotonic glycoside that may exist in AAS were detected by UPLC-Q-Orbitrap HRMS. Some scholars reported that oleanolic acid protects VSMC injury by activating AKT/eNOS signaling [56] or NLRP3 inflammasome signaling pathways [57]. There are also scholars that reported that digoxin inhibits PDGF-BB-induced VSMC proliferation and migration through an increase in ILK signaling and attenuates neointima formation following carotid injury [58]. The above conclusions indicate that saponins are most likely to be the main components of AAS to protect against vascular damage. However, which saponins are specifically effective and what is the mechanism of action have not been resolved. Therefore, further studies are necessary to identify the active compounds and their potential molecular mechanisms.

## 5. Conclusion

Our data indicate that *Aralia armata* (Wall.) Seem (AAS) can reduce the pathological changes of restenosis after vascular injury by downregulating the Wnt pathway. We suggest that AAS can be beneficial for patients undergoing PTCA or stent implantation.

## Figures and Tables

**Table 1:** Primer/probe sequence for real-time RT-PCR.

Target gene	Sequence	
Wnt3*α*	Forward primer	5′-CCCTCGGAGCCCGTGTA-3′
	Reverse primer	5′-ACCACCAAATCGGGTAGCTG-3′
*β*-Catenin	Forward primer	5′-CTGCTGATCTCGGACTGGAC-3′
	Reverse primer	5′-GTCGGTATCAAACCAGGCCA-3′
Gsk-3*β*	Forward primer	5′-GAGACACACCTGCCCTCTTC-3′
	Reverse primer	5′-TGGGGCTGTTCAGGTAGAGT-3′
Dvl-1	Forward primer	5′-GAGCTGGGACTACCTCCAGA-3′
	Reverse primer	5′-AGTGGTGCCTCTCCATGTTG-3′
Cyclin D1	Forward primer	5′-TCAAGTGTGACCCGGACTG-3′
	Reverse primer	5′-CACTACTTGGTGACTCCCGC-3′
GADPH	Forward primer	5′-AGTGCCAGCCTCGTCTCATA-3′
	Reverse primer	5′-ACCAGCTTCCCATTCTCAGC-3′

## Data Availability

The data used to support the findings of this study are available from the corresponding author upon request.
